# Statistical Analysis of Bathing Water Quality in Puglia Region (Italy)

**DOI:** 10.3390/ijerph15051010

**Published:** 2018-05-17

**Authors:** Daniela Malcangio, Claudio Donvito, Nicola Ungaro

**Affiliations:** 1 DICATECh, Polytechnic University of Bari, Via E. Orabona n.4, 70125 Bari, Italy; 2 Freelance, Largo G. Ungaretti 2, 70056 Molfetta, Italy; claudio.dnv@gmail.com; 3 ARPA Puglia, Corso Trieste 27, 70126 Bari, Italy; n.ungaro@arpa.puglia.it

**Keywords:** spatial and temporal analysis, Indicator Kriging, bathing water quality indicators, faecal contamination, coastal water monitoring

## Abstract

Geostatistic analysis was applied to the dataset from multi-year monitoring, in the Apulian marine-coastal zone (Mediterranean Sea, Italy), on the presence and abundance of intestinal Enterococci and *Escherichia coli*, microbiological indicators of faecal contamination at the sea. The same faecal contamination can be considered as the main cause of pollution phenomenon under current Italian and European regulations for the bathing waters (Italian Government Decree 116/2008, European Directive 2006/7/CE). The main objective of the study is to verify, taking into the account the anthropic pressures acting on the coastal zone, the efficiency of the Apulian regional monitoring plan currently in force for the assessment of bathing waters quality, with a view to a hypothetical reduction of sample collection points.

## 1. Introduction

The Italian coastline stretches for more than 8000 Km, making it the European country with the highest number of bathing waters, 5518 (about a quarter of the European total), of which 4866 are marine-coastal bathing waters and 652 are inland bathing waters (rivers and lakes). The national coastline is highly anthropized, featuring intensive urbanization (houses and infrastructures, 58%) and extensive settlements (13%). Under Italian law, bathing waters management and monitoring is regulated by the Government Decree n. 116/2008 in compliancy with European Directive 2006/7/CE establishing the objectives of bathing quality management [1]. Inspired by the most recent scientific knowledge, the Directive issued by the European Parliament in 2006 suggests an integrated and innovative approach, including bathing water profile, four quality categories for bathing waters classification, short-term pollution forecast and public participation.

At national level and in compliancy with the above Directive, the Italian Government Decree 116/2008 is aimed at safeguarding human health from risks that may derive from bathing in poor quality waters also by means of environment protection and enhancement. To such purpose, the decree establishes how to monitor, classify, manage and diffuse information on the quality of bathing waters. Under the new regulation, the criteria for the definition and classification of bathing waters are essentially based on the concepts of forecasting and evaluating health risks, with less emphasis on temporary bathing suitability conditions.

A few human pathogens can be ascribed to autochthon microorganisms from a water environment (*Legionella*, *Vibrio*, *Shigella*). The majority of these pathogens are due to particular microorganisms (*Salmonella*, gastroenteric viruses) deriving from the gastrointestinal tract of humans or warm-blooded animals, as a consequence of faecal contamination of the water environment. The concentration of these pathogens highly depends on the efficiency of waste water collection and treatment systems as well as from the self-purification ability of the receiving water bodies, often favoured by natural factors such as the presence of current [2,3] or vegetation [4,5]. Surveying and quantifying faecal microorganisms can be quite expensive and complicated, therefore the evaluation of water microbiologic quality is generally based on the identification and study of specific organisms to be used as “indicators”.

In order to be considered as an indicator, an organism must: (i) be present in the water every time pathogen microorganisms are present; (ii) have a life-span similar to the life-span of pathogen microorganisms; (iii) have a resistance to depuration and disinfection treatments similar to the resistance shown by pathogen microorganisms; (iv) its characteristics should not change over time.

Establishing the best bacteriological indicator system for water quality monitoring has historically been a significant issue which already occupied a great deal of time of the American Public Health Association committees in the 1930s and continued to feed a debate through the 80s, with interesting results [6]. The main microorganisms historically used as indicators of water’s faecal contamination are total coliforms, faecal coliforms and faecal streptococci. By effect of the new European regulation, only two microbiological parameters are defined as anthropic pollution indicators: intestinal Enterococci (IE) and *Escherichia coli* (EC). They are the most widely-used faecal indicator bacteria [7], which have added acceptance in health warning systems because of their verified relationship to risk of illness [8]. Each indicator is expressed in terms of a count of colony forming units per 100 mL (CFU/100 mL), but there are also other accepted and often used standard methods so that the density can be based on most probable numbers (MPN) or genetic methods like PCR. The new European regulation also impacted on sampling frequency and analysis parameters, establishing that monitoring should be exclusively carried out in bathing water. In Italy, the Government Decree 116/2008 provides for a monthly sampling frequency, between April and September. Cheung et al. [9] conducted a study on the daily and hourly variations in microbial indicators densities in the beach-waters of Hong Kong. They found that the variability of microbial indicator densities that means the routine sampling of bathing beaches should be carried out on weekend days with maximum numbers of swimmers exposed to the water [9]. More recently, Griffith et al. [10] highlighted the need to establish that new indicators measured by molecular methods are equal or better predictors of health risk than are existing indicators. Their results from epidemiologic studies in three Californian beaches, during which a series of alternative indicators were monitored simultaneously with *Enterococcus* (measured by Environmental Protection Agency Method 1600), suggested that site-specific conditions at each beach determine which indicator or indicators best predict health risk. Confirming what just stated, Molina et al. [11] identified environmental parameters, as alternative faecal indicators and microbial source tracking (MST) markers (including human-associated), and pollution sources that can influence the concentration and transport of molecular faecal indicator bacteria in systems solely impacted by urban runoff.

Consequently, the use of organisms as “indicators” does not allow a direct estimate of the presence of a given pathogen microorganism in the water environment, yet it allows to forecast the probability of its presence, therefore verifying the quality of bathing water. The Italian Government. Decree n. 116 issued on 30 May 2008 and its subsequent amendments established the IE and EC concentration limits to allow bathing at 200 CFU/100 mL and 500 CFU/100 mL, respectively. The subsequent classification is based on a quality evaluation obtained by means of a statistical calculation (95th percentile—or 90th percentile—evaluation of the normal function of log10 probability density of microbiological data). Such evaluation is carried out every year at the end of the bathing season based on the data available for that season and compared with three previous seasons. It distinguishes four quality categories based on the microbiological indicators’ density, as shown in Table 1. The results of such classification can be used to define quality percentages at regional and provincial scale. In pursuance of the Directive in force, when bathing water is temporarily classified as “poor” quality water, citizens should be promptly informed, and the concerned authorities should apply specific management measures, such as prohibiting or advising against bathing in order to prevent microbiological contamination risks for bathers. If a “sufficient” quality standard hasn’t been met, measures are also required to investigate, reduce or eliminate the pollution causes. If bathing water quality is classified as poor for five consequent years, bathing prohibition shall become permanent.

Test frequency may vary, based on the pollution type and significance, in compliancy with the terms indicated in Table 2. Previous works on this subject in literature have tried to verify the effectiveness of the European Directive at local scale. Chawla and Hunter [12] and Chawla et al. [13] tried to put the Bathing Water Directive into practice before its publishing, applying the proposed method of using parametric percentile values to assess the classification of Irish bathing water sites.

They concluded that the method to gauge compliance was statistically unreliable due to failure of the log-normality assumption at many beaches. More recently, this assumption was investigated by Haggarty et al. [14] for the Scottish bathing waters. Their analysis indicated that overall there was general agreement between the parametric and empirical based classification of each site, with only a few exceptions at sites, which lie on the border of two classification categories. Moreover, they suggested a method of obtaining limits, which identify the samples that should be removed from compliance calculations, as well as to take care of the quantity of data that should be removed.

Scientists have always tried to study and comprehend the dynamics of natural and man-induced phenomena, such as costal water pollution, so to be able to predict and control them. These phenomena come in a wide and variegated range: from the simplest, showing in space and characterised by a single variable, to the most complex ones, ruled by a great number of mutually interacting variables that can be defined in space as well as in time [15].

Studying such complex natural phenomena has required the development of specific mathematical and statistical instruments for both field monitoring and data analysis. A continuous and real-time field monitoring allows not only to assess water environment quality, but also to prevent its deterioration, since it allows collecting data at large time and space scales. In the last 30 years, several video system techniques, based on image processing, have been developed in order to extract from images different information e.g., [16,17]. At the same time, geostatistics has become widely applied in the field of environment and territorial data treatment [18]. One of the most useful and popular geostatistical method in many fields is kriging. This is a complex technique of interpolation, which is the process of filling gaps between sample observations to produce a grid of values [19]. There are plenty of interpolation methods, e.g., linear regression, nearest neighbour, inverse distance weighting, spline (a polynomial function) and kriging. The latter has proven a good choice where the sample points are poorly distributed or there are few of them [20]. This means that it encompasses autocorrelation (i.e., everything is related to everything else, but near things are more related than distant things). This statistical relationship between the measured points enables estimation errors (kriging variance) to be reckoned [21].

On account of its widespread use, suitability for the data and error calculations, one form of kriging, i.e., Indicator Kriging (IK), is used to process data in this research. The Indicator Kriging (IK) technique allows evaluating the probability of exceeding the established limits at interpolation points. For some sites, sampling areas grouping procedures were also studied, testing a reduction of monitoring points to verify whether such reduction, with its significant political and environmental effects, may interfere with the interpolation activity previously analysed.

## 2. Materials and Methods

### 2.1. Geostatistics and Indicator Kriging Estimation Method

The application of geostatistic analysis aims at the spatial or temporal characterisation of data and information as well as at their modelling and estimate [22,23]. Besides offering an objective representation for the interpretation and comprehension of phenomena, the results obtained from geostatic processing can be useful in decision-making as well as in the development and management of monitoring plans.

The spatial, temporal or spatial-temporal characterisation of a phenomenon is the first step in a geostatistic study. It essentially consists in highlighting, in terms of quality and quantity, the phenomenon variability, specifying its typology in relation to the presence of any spatial-temporal anisotropies and variables [24,25].

Once the phenomenon characterisation has been completed, variables can be spatially reconstructed by estimate. This stage consists in the spatial reconstruction of a variable, starting from a limited number of measures of the same variable [26]. If need be, it is possible to integrate measures of related auxiliary variables which can be more easily and less expensively acquired [27].

Estimating a variable is most commonly done as part of spatial data processing. It aims at constructing thematic maps, i.e., geo-referenced maps of a portion of a geographic area, showing the spatial-temporal trend of an interesting variable by means of a suitable representation method [28].

The method used for estimations under this study is the Indicator Kriging (IK), a linear indicator that can be applied to data characterised by non-Gaussian distribution, similar to ordinary Kriging but with the input of a threshold value [29].

It does not use its own values of the variable to be estimated, but rather it makes use of new variable indicators to obtain a prediction on the values. Unlike ordinary Kriging, the IK is a type of non-parametrical interpolation and can estimate a value at an unknown point by means of the aggregate distribution function reconstructed using the thresholds.

If we name the region that contains all ***N*** observations available as ***D*** ⊂ R^2^, then for each ***x*** ⊂ ***D***, taken a value of the searched field characteristic as threshold ***z***, the following indicator function can be defined:

(1) $$i\left(x;z\right)=\{\begin{array}{c} 1\;if\;z\left(x\right)\leq z\\ 0\;if\;z\left(x\right)>z\; \end{array}$$

The objective of this approach is to provide a prediction of the regionalized variable ***z***(***x***) for any location ***x*_0_** within the examined area ***D***, given the ***N*** available observations.

The estimated value in point ***x*_0_**, determined based on the indicator values (***x*_0_**; ***z_k_***), which depend on the selected cutoff value ***z***, is defined by the formula:

(2) $$\hat{I}\left(x_{0};z_{k}\right)=\overset{n}{\underset{i=1}{\sum }}w\left(x_{i};z\right)I\left(x_{i};z\right)-\left[1-\;\overset{n}{\underset{i=1}{\sum }}w\left(x_{i};z\right)\right]F\left(z\right)$$

where $w\left(x_{i};z\right)$ are the weights of kriging.

This value may be presented as the value of the cumulative distribution function of the regionalized variable ***z***(***x***) for the cutoff value ***z_k_*** in point ***x***_0_:

(3) $$\hat{I}\left(x_{0};z_{k}\right)=E\left\{\left[x_{0};z_{k}|\left(n\right)\right]\right\}$$

where ***n*** is the conditioning caused by the proximity of other values at point ***x*_0_**.

The conditional cumulative distribution function of the regionalized variable ***z***(***x***) for any location within the examined area, at the cutoff value ***z_k_***, will be:

(4) $$\hat{I}\left(x;z_{k}\right)=E\left\{\left[x;z_{k}|\left(n\right)\right]\right\}=Prob\left\{x)\leq z|\left(n\right)\right\}$$

An accurate description of the IK and its comparison with other estimation methods are respectively well discussed in Journel [30,31] and Goovaerts [32,33].

Indicator kriging allows the preparation of maps of the probability with which the regionalized variable ***z***(***x***) takes specific values, limited by any given cutoff values, which is extremely important in practical use, e.g., to evaluate the impact of contaminants on water quality. In this case, geostatistical analysis can be useful both for definition of bathing water profile and the evaluation of effects due to “short term pollution” and “abnormal situations”. The application of IK can provide useful elements to identify the extent of the area being affected by pollution, as well as to estimate whether the pollutant load may represent a health risk. Therefore, it is a valid instrument to formulate and evaluate the effectiveness of environmental management measures.

### 2.2. Case Study

This work was carried out with the objective of studying and analysing the phenomenon of marine water pollution deriving from faecal contamination at local scale, using as study area a region in Southern Italy, Puglia, with the aim of detecting any critical areas along its coastline.

The regional coastline of Puglia stretches close to 1000 km (Regional Coastal Plan 2011), ranking third in length at national level after only Sardinia and Sicily. The geomorphology of the Apulian coastline varies as we move along the coast, featuring sandy beaches, river mouths as well as rocky cliffs. Such a natural coastal variety is emphasised by the effects of anthropic development (e.g., urbanization, population density, infrastructures), which impact on the marine-coastal ecosystem.

**Figure 1:**
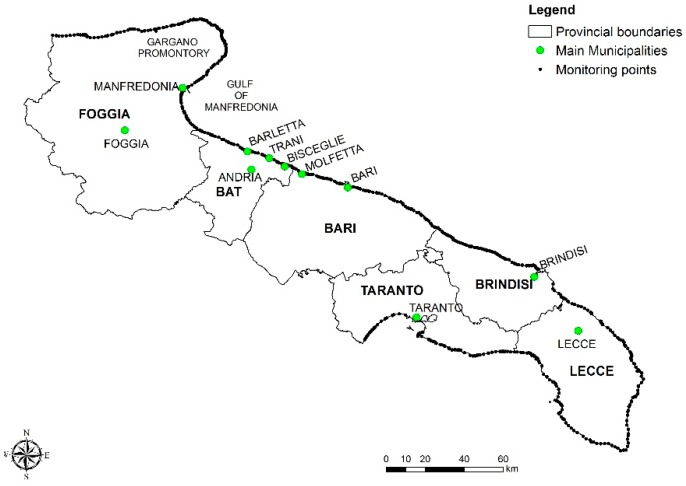
Map of monitoring points.

In compliance with national and European regulations, 674 different bathing waters have been identified in the region, according to the water basins, hydrological and morphological characteristics and the anthropic pressures along the coastline. Each of bathing waters is monitored in pursuance of Government Decree 116/2008, which establishes the rules for such activity in Italy. The data used for this study were provided by the Regional Agency for Environmental Prevention and Protection (ARPA Puglia), responsible for bathing water control on behalf of the Puglia Region-Department of Health, in compliancy with the national Government Decree 116/2008. The data were collected from the “Bathing Waters Monitoring Bulletin” issued by ARPA Puglia for the years 2010, 2011, 2012, 2013 and 2014 (http://www.arpa.puglia.it/web/guest/balneazione) and refer to monitoring activities carried out at a monthly frequency between April and September each year along the Apulian coastal zone. Figure 1 shows all the monitoring points, together with the main municipalities mentioned in the text that follows.

## 3. Results

### 3.1. Data Set Analysis

The monitoring data were first structured and ordered on the basis of the bathing water denomination, the bathing area code, the municipal belonging, the position and Gauss-Boaga geographical coordinates and, lastly, the numerical values of the microbiological contamination detected (ordered by sampling data and microbiological indicator). The microbiological parameters measured are expressed in Colony Forming Units per 100 mL of water (CFU/100 mL). The total number of records available for this study was 18,360.

**Figure 2:**
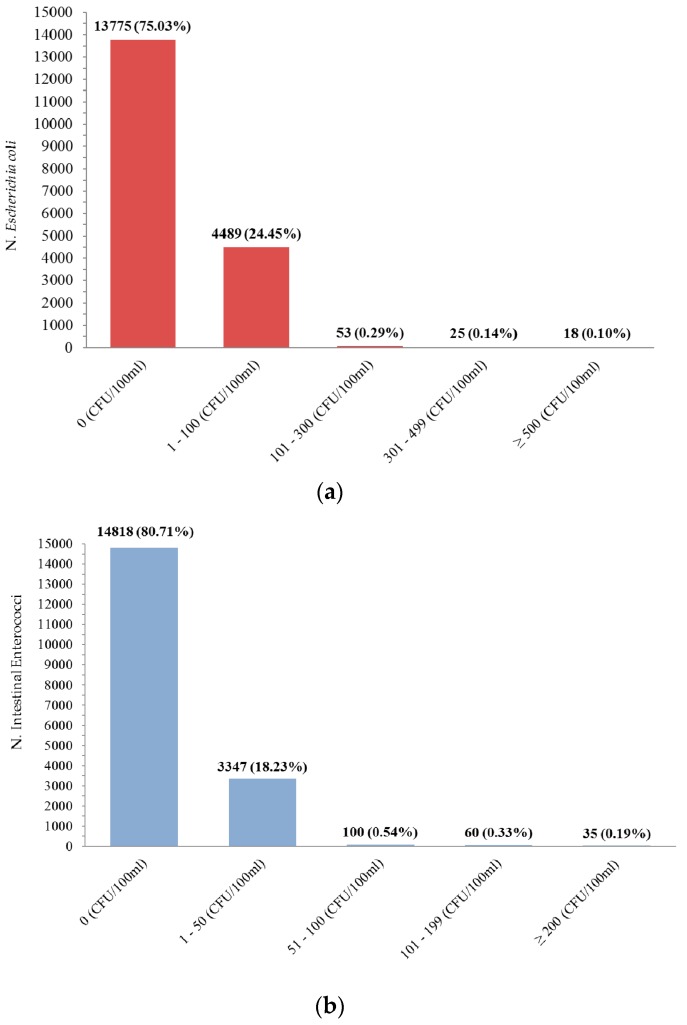
Concentration ranges in the study area of *Escherichia coli* EC (**a**), Enterococci IE (**b**).

**Figure 3:**
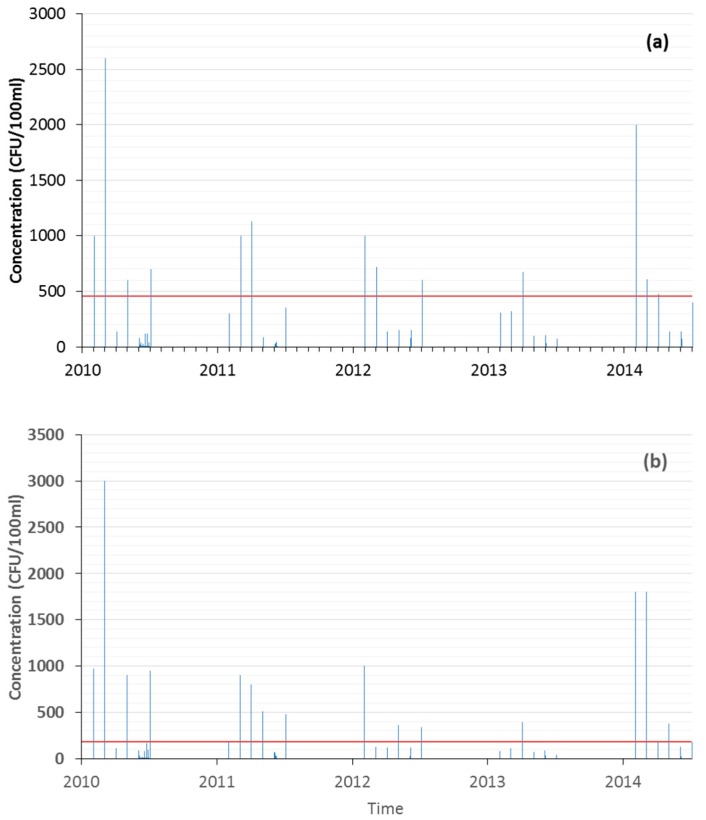
Temporal evolution of maximum concentration values of (**a**) EC, (**b**) IE. Red horizontal line refers to threshold of “good quality”.

The distribution of EC and IE data showed the prevalence of null values and a small number of records over the threshold values established under the regulation in force for bathing suitability Figure 2. The temporal trend of maximum values was also represented for both EC and IE microbiological parameters Figure 3. Such analysis highlighted most of the maximum reported values during April and May in almost all the years under study.

### 3.2. Geographic Information System (GIS) Analysis

The GIS processing of EC and IE Colony Forming Units analytical results was performed after categorization of data according to fixed intervals see Figure 2.

**Figure 4:**
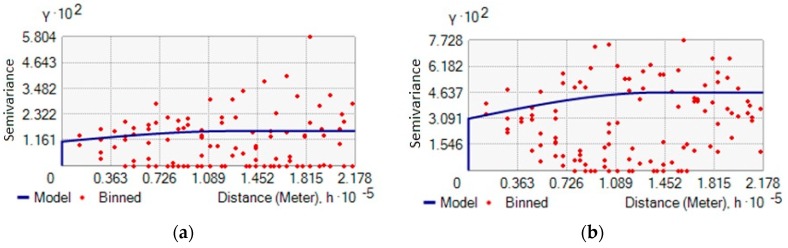
Variogram of EC data (**a**), IE data (**b**).

The geostatic technique used for prediction under this study is the Indicator Kriging (IK), a linear interpolation similar to ordinary Kriging with the input of a threshold value. This technique provides a value for the probability that the threshold established is overcome at interpolation points. The spatial distribution of threshold overcoming probability was achieved taking into account the values shown in the variograms of Figure 4.

Table 3 shows the spatial analysis parameters and Root Mean Square Standardized (RMSS). The latter should be as close as possible to 1 to obtain a correct interpolation, therefore 1.051 and 0.9228 are acceptable values. The spherical semi-variogram model was chosen due to very low Nugget values, i.e., 0.01 for EC and 0.03 for EI. The Lag number for both microbiological parameters was 12, with a Lag size of about 18,000 m. The Range value beyond which there is no longer spatial correlation among data was 125,988 m for EC and 141,775 m for EI.

**Figure 5:**
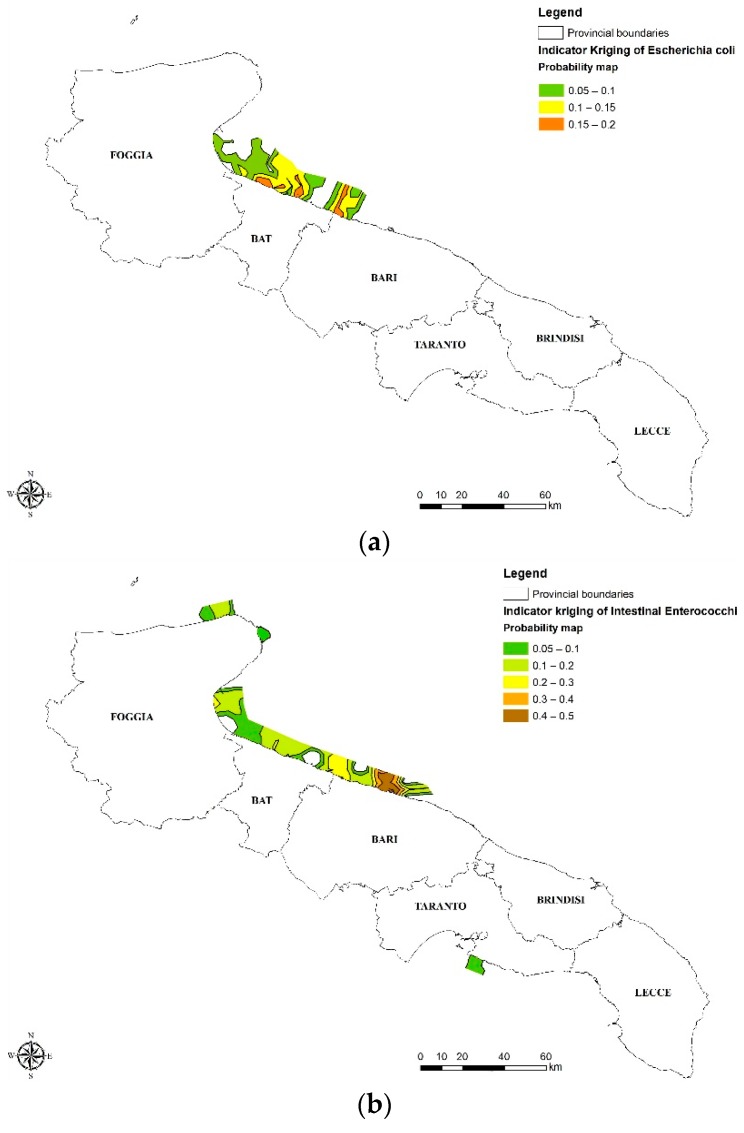
Map of threshold overcoming probability for EC (**a**), IE (**b**).

The probability values observed were mapped in Figure 5 using ArcGis software to obtain a direct image of the threshold overcoming probability of spatial distribution. The selection of thresholds was carried out in consideration of “good quality” limits established by the regulation in force, i.e., 500 CFU/100 mL for EC and 200 CFU/100 mL for IE. The highest threshold overcoming probability for EC Figure 5a was observed in the area between the Northern territory of Bari and the Gulf of Manfredonia, showing a probability of exceeding the threshold that reaches 20%. For the IE, the probability values obtained were found to be greater than the EC indicator, with a maximum value of 50% near the city of Bari Figure 5b. Moreover, the analysis carried out shows the threshold overcoming probability for EI extended for a larger area, up to the Gargano promontory and the city of Taranto Figure 5 below. Both probability maps in Figure 5 indicate that the problem is geographically localized, as the most affected areas are always the same. This implies a specific criticality, which may lead to a chronic phenomenon in these areas.

**Figure 6:**
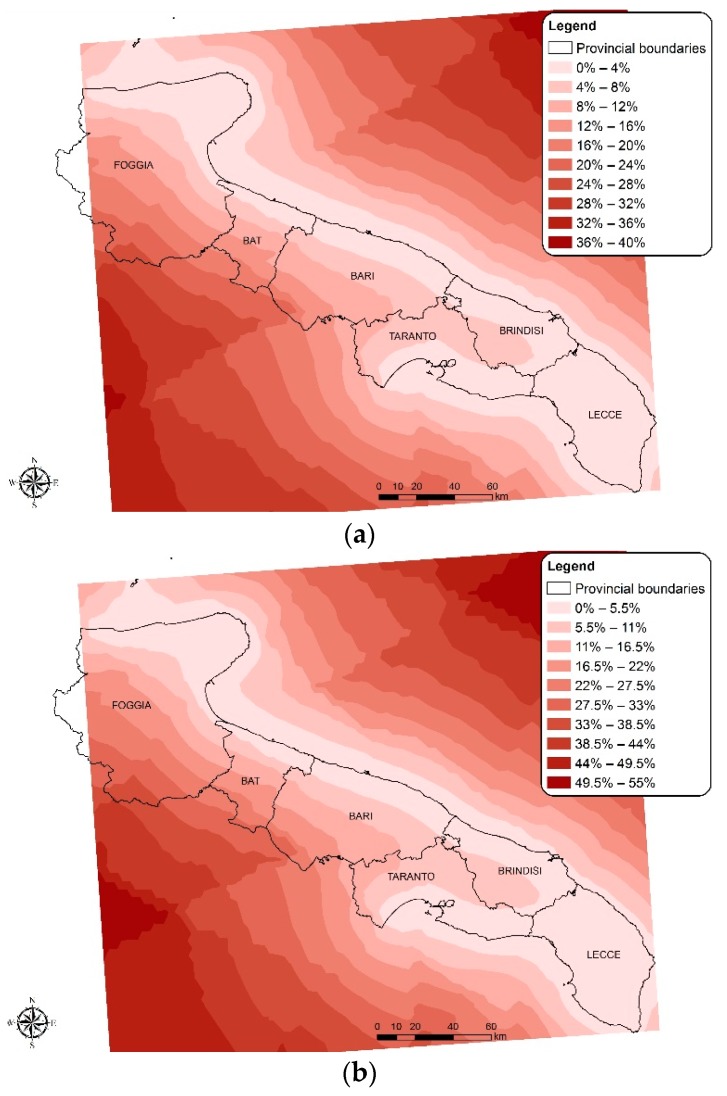
Map of error probability for EC (**a**), IE (**b**).

The municipalities of Molfetta, Bisceglie and Bari are the biggest concerns, since they show a high threshold overcoming probability from contamination for both microbiological indicators. The software used also provides an estimate of error probability (in the form of error variance). Such estimate can be utilised for a direct evaluation of prediction reliability. As shown in Figure 6, the percentage error for the area closest to the coastline was between 0% and 5.5%. The percentage error increases moving away from the coast, both towards the land and towards the open sea. This means that the point data available were numerically sufficient for the classification of water quality.

**Figure 7:**
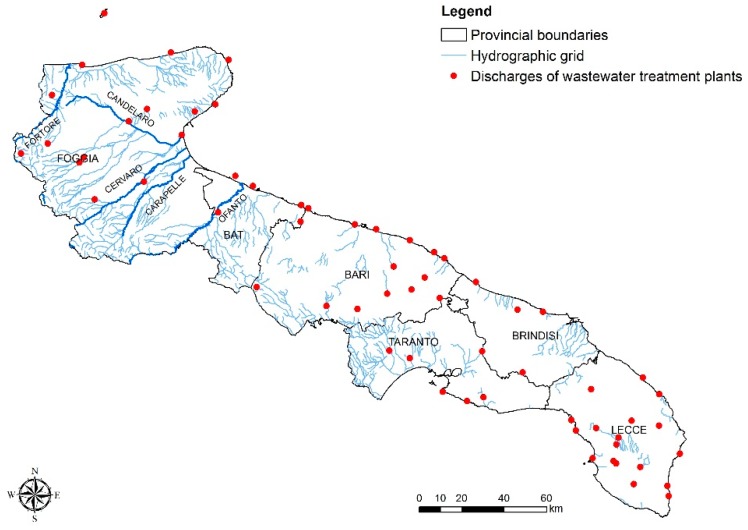
Regional hydrographic network with discharge points in Puglia.

A range of thematic maps were also developed in order to establish the correlation between the results obtained and the point or diffused anthropic pressures. Figure 7 shows the distribution of discharge points of waste water treatment plants in the Apulian territory, together with the regional hydrographic network. The implementation of information related to the river network represented in Figure 7 provides significant details on the relation between waste water discharging in inland waters (rivers and lakes), or on the ground, and those directly discharging in marine-coastal waters. As far as the Apulian rivers network is concerned Figure 7, the main EC and IE contamination can be observed in the coastal water along the Northern part of the region Figure 5. This result denotes the potential correlation between waste water discharge into inland water bodies and the quality of coastal waters [34,35]. A correct resizing of treatment plants may provide a possible solution for improving depuration processes and reducing the sea pollution. Particularly in the case of Puglia, if not available other valid options in terms of environmental sustainability (i.e., reuse of treated water for other uses), a relocation of some discharges at sea shall be taken into consideration, as well as planning new submarine pipes or the lengthening of existing ones, to allow a better dilution and distribution in the sea of waste treated waters and avoid their potential impact on the coastal zone.

**Figure 8:**
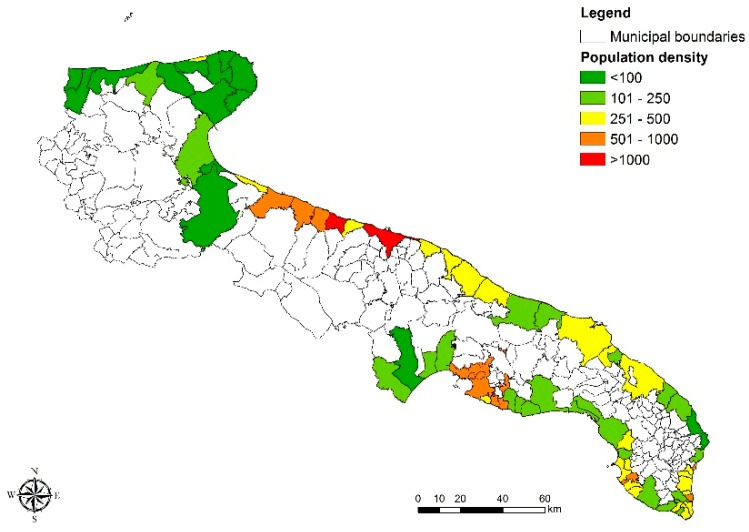
Coastal population density as km^2^ in Puglia.

The last analysis was carried out based on the coastal population density in terms of inhabitants/km^2^. The information on the territorial extension of Apulian coastal municipalities was obtained from the Apulian Territorial Information System website (SIT Puglia), while data on the number of inhabitants were collected from the 2011 population census dataset published by the Italian National Institute of Statistics (Istat). Looking at Figure 8, it can be easily assumed a relation between the highest faecal contamination values and the highest population density of coastal municipalities, e.g., Bari and Molfetta. This evidence confirms that population density alone can be one of the most important factors affecting the bathing waters pollution from faecal contamination.

### 3.3. Bathing Water Grouping Procedure Analysis

As established under the current Italian regulation, in some cases contiguous bathing waters within the same water basins can be grouped into a unique bathing water, if under the same anthropic pressure and health risks. Thus, a purely qualitative grouping procedure was simulated, grouping contiguous bathing waters maintaining the highest measured values of contamination for the widest areas. For the purpose of this work, the analysis was carried out at different scales, taking into account two specific areas along the Apulian coastline, featuring a critical but different extent of pollution from IE faecal contamination, i.e., the sea coastal areas under the municipal territories of Molfetta and Bari.

**Figure 9:**
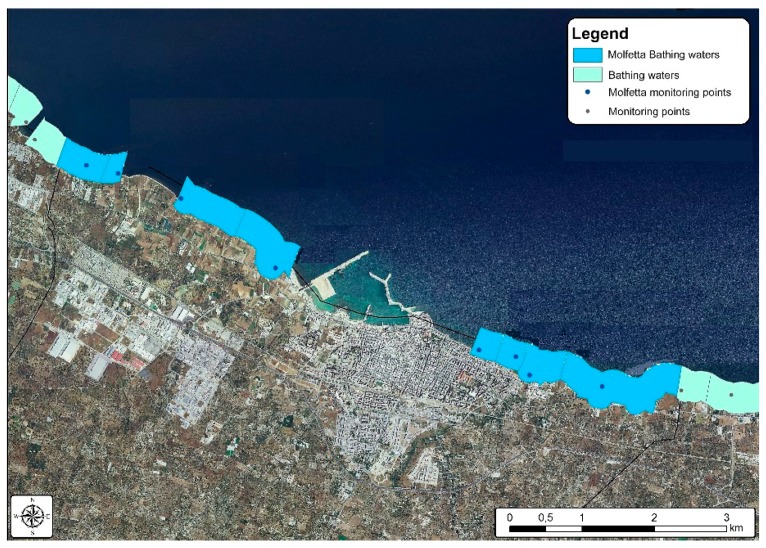
Bathing waters in the municipality of Molfetta.

**Figure 10:**
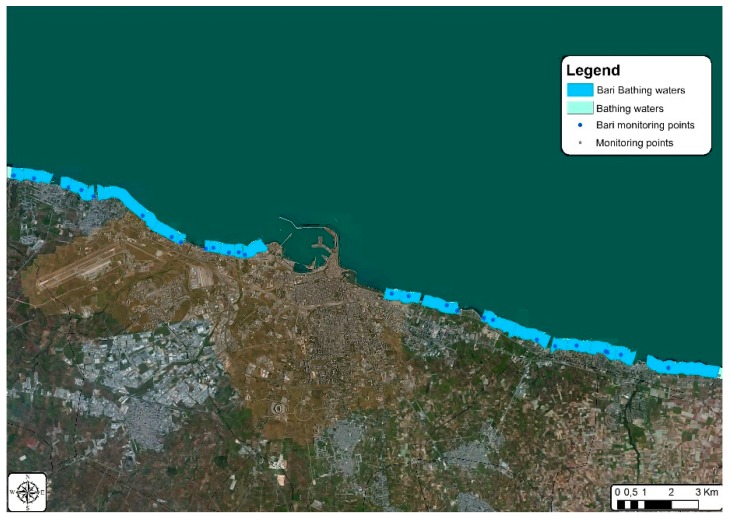
Bathing waters in the municipality of Bari.

Figure 9 and Figure 10 show the location and subdivision of the bathing waters as currently defined: in the municipality of Molfetta there are eight bathing waters, four to the West and four to the East of the harbour; a higher number of bathing waters is defined for the municipality of Bari, with a total of twenty-six.

**Figure 11:**
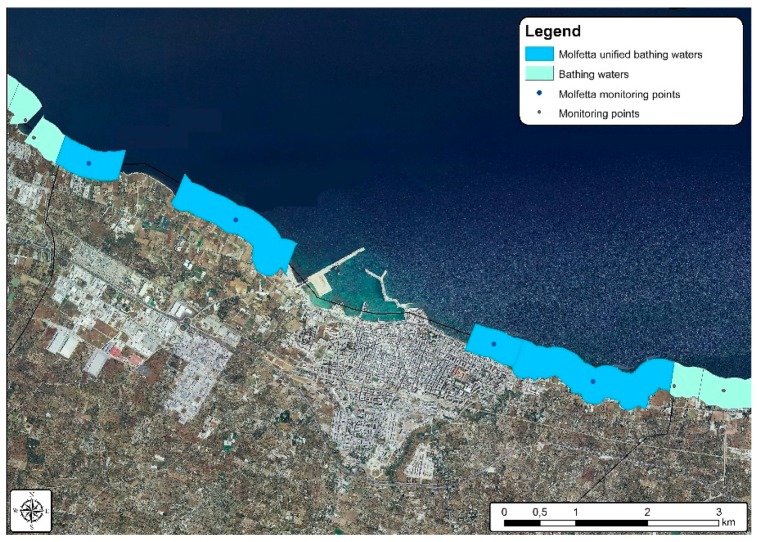
Bathing waters in the municipality of Molfetta after the grouping procedure simulation.

**Figure 12:**
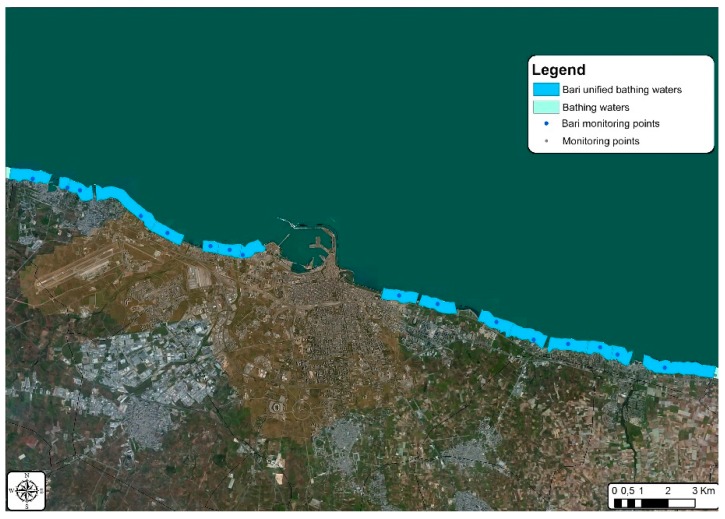
Bathing waters in the municipality of Bari after the grouping procedure simulation.

The grouping procedure simulation was carried out by halving the number of bathing waters in the municipality of Molfetta from eight to four Figure 11, while a 38% reduction was applied for the municipality of Bari, from twenty-six to sixteen Figure 12.

**Figure 13:**
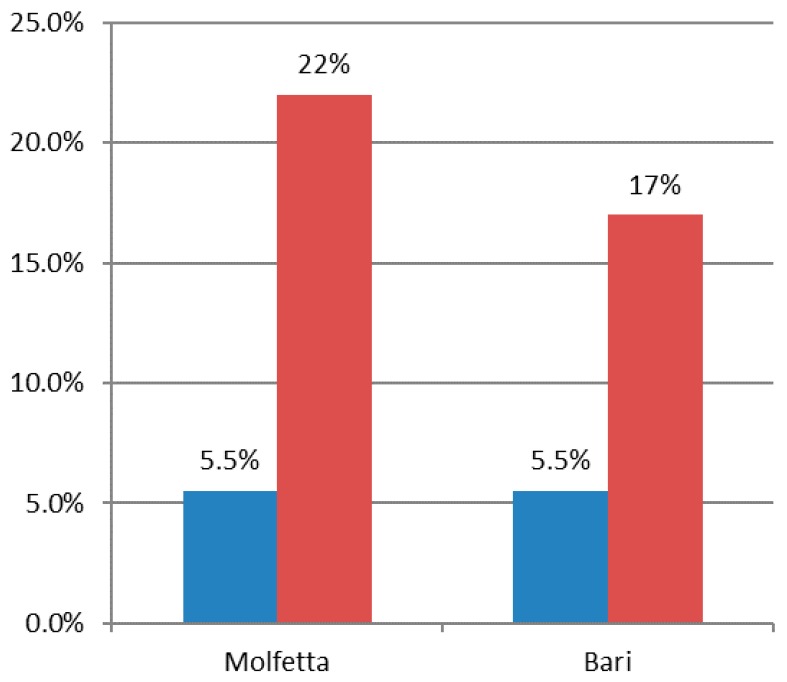
Minimum probability of error before (blu, left) and after (red, right) the grouping procedure simulation.

Upon completing the grouping procedure simulation, the IK spatial interpolation method was applied, using data exclusively related to IE concentration. Such process produced the same typology of results above described, with very similar probability values but with significant variation in terms of minimum error probability, with consequent impact on prediction reliability. Figure 13 shows an increase from about 5% to 17–22% minimum error probability for both the municipalities of Bari and Molfetta. The difference in the error probability percentage between the two municipalities can be mainly ascribed to the different reduction percentage applied to the number of their monitoring points.

This analysis shows, in the case of the Apulian coastal area under study, that a grouping procedure and therefore a reduction in the actual number of related monitoring points may determine a higher error probability in the evaluation of water quality. The spatial scale used in this type of evaluation can significantly impact on the final result, too.

## 4. Conclusions

This research confirmed the IK as a good tool for spatial interpolation of environmental data, ordered on the basis of the bathing water denomination, the bathing area code, the municipal belonging, the position and Gauss-Boaga geographical coordinates and, mainly, the numerical values of the microbiological contamination detected along the Apulian coasts.

The study highlights the potential correlation between waste water discharge into inland water bodies and the quality of Apulian coastal waters. Discharge systems carrying waste waters to the sea through a river can contribute to an increased faecal contamination risk along the coast, although some biological constrains have to be considered (i.e., the half-life or the “log10-reduction” for the elimination of enterobacteria populations in the sea). The overlapping between the contamination maps and the coastal inhabitants’ one underlines that population density alone can be one of the most important factors affecting the bathing waters pollution from faecal contamination. So, the importance of the anthropic pressures assessment was highlighted, since strictly related to the results obtained.

Moreover, data processing qualitatively showed that grouping Apulian bathing waters and therefore reducing monitoring points determine a not appropriate evaluation, with a significant increase of the minimum error probability, of bathing water quality, demonstrating the congruity of the actual monitoring plan implemented by the regional agency ARPA Puglia. It is necessary to consider also spatial scaling as a factor that may considerably modify final results in the sampling point optimisation process.

This study indicates the possibility to process historical data to obtain worthwhile information for the assessment of water monitoring plans, as well as for studying pollution phenomena at relevant spatial scale. The methodology discussed turned out to be useful to the concerned authorities for the planning of environmental impact mitigation measures, as well as for the improvement of sectorial regulations.

## Figures and Tables

**Table 1:** Quality classification of (**a**) inland waters, (**b**) coastal and transitional waters, by Government Decree 116/2008.

Parameter	Excellent	Good	Sufficient	Reference Methods of Analysis
(**a**)				
Intestinal Enterococci (CFU/100 mL)	200 (*)	400 (*)	330 (**)	ISO 7899-1 or ISO 7889-2
*Escherichia coli* (CFU/100 mL)	500 (*)	1000 (*)	900 (**)	ISO 9308-3 or ISO 9308-1
(**b**)				
Intestinal Enterococci (CFU/100 mL)	100 (*)	200 (*)	185 (**)	ISO 7899-1 or ISO 7889-2
*Escherichia coli* (CFU/100 mL)	250 (*)	500 (*)	500 (**)	ISO 9308-3 or ISO 9308-1

(*) Based upon a 95-percentile evaluation, (**) Based upon a 90-percentile evaluation.

**Table 2:** Sampling frequency, by Government Decree 116/2008.

Bathing Water Classification	Good	Sufficient	Poor
Surveys are to take place at least every	four years	three years	two years

**Table 3:** Parameters of the spatial distribution of the threshold values exceedance probability.

Parameter	Model	Range (m)	Nugget	Partial Sill	RMSS *
***Escherichia coli***	Spherical	125,988	0.01	0.0047	1.051
**Intestinal Enterococci**	Spherical	141,775	0.03	0.0154	0.9228

* RMSS: Root Mean Square Standardized.
